# From silence into song: an art–science collaboration with survivor trees and laryngectomy singers

**DOI:** 10.3389/fpsyg.2025.1747218

**Published:** 2026-01-30

**Authors:** Thomas Moors, Evangelos Himonides

**Affiliations:** 1 Department of Art History, Institute for Psychoacoustics and Electronic Music (IPEM), Musicology and Theatre Studies, Ghent University, Ghent, Belgium; 2 Otolaryngology, Lancashire Teaching Hospitals NHS Foundation Trust, Preston, United Kingdom; 3 Shout at Cancer, London, United Kingdom; 4 University College London, London, United Kingdom

**Keywords:** arts-in-health, co-creation, ecological metaphor, laryngectomy, qualitative research, radiotherapy, reflexive thematic analysis, survivor trees

## Abstract

**Introduction:**

This paper presents an immersive art–science project that unites nature, voice, and technology to examine the dual role of radiation as both a force for destruction and a means of healing, through the experiences of two survivor communities: Hibakujumoku (trees that survived the atomic bombings of Hiroshima and Nagasaki) and individuals who lost their voices to head and neck cancer and rebuilt communication through radiotherapy, surgery, and rehabilitation.

**Methods:**

Ten adults, post-laryngectomy, were recruited via Shout at Cancer, a UK charity focused on alaryngeal speech recovery. Over ten weeks, they attended six workshops combining group singing, creative writing, and reflective dialogue. Participants listened and responded to recordings of survivor trees from Japan, captured with contact microphones, accelerometers, and hydrophones. Infrared and thermal imaging revealed hidden vitality. These materials, integrated with participant vocal recordings, formed hybrid works presented as live performances and multimedia installations. Analysed data comprised workshop audio recordings and notes, participant reflections, creative texts where functioning as reflective accounts, researcher field notes and reflexive memos, and written reflections from collaborating artists. Data were analysed using reflexive thematic analysis within an interpretivist, arts-based participatory design.

**Results:**

Reflexive thematic analysis identified three themes: (1) *Parallel Survivorship*—encounters with trees’ “voices” prompted awe and reframed silence as endurance; (2) *Reclaimed Agency*—co-writing and performance supported identity, confidence, and public presence; (3) *Collective Embodiment*—shared vocal practice with human and non-human sounds fostered synchrony, joy, and social connection. Reflections from collaborating artists described reciprocal change, noting shifts toward listening, service, and shared authorship.

**Discussion:**

Where radiation carries complex cultural meanings, these findings highlight the importance of reframing it within clinical and public health contexts—as both a source of harm, and a means of healing. The results demonstrate that immersive, nature-linked co-creation not only assists in meaning-making and relational wellbeing for individuals recovering from voice-altering cancer treatments, but also underscores the potential of such approaches to complement healthcare interventions by fostering emotional recovery and social connectedness. The study furthermore strengthens the existing underpinnings for future mixed-methods and longitudinal research to examine the broader impacts of arts–health collaborations.

## Introduction

Over the past two decades, societies have faced crises such as pandemics, environmental degradation, and geopolitical conflict. These events have sharpened attention on resilience, wellbeing, and how communities adapt to disruption (Masten et al., 1990; Seery et al., 2010). Research in arts-and-health and environmental psychology shows that creative engagement and meaningful contact with nature can support emotional regulation, social connection, and recovery following adversity (Kaplan and Kaplan, 1989; Stuckey and Nobel, 2010; Fancourt and Finn, 2019).

This study presents an interdisciplinary art–science project that connected two survivor communities affected by radiation: Hibakujumoku, trees that survived the atomic bombings of Hiroshima and Nagasaki, and people who lost their natural voice after head and neck cancer treatment including radiotherapy and laryngectomy. The project focuses on the paradox that radiation is both destructive and therapeutic. We explored how this dual legacy could be re-narrated through participatory artistic practice.

Exposure to natural environments has been shown to restore attention and support wellbeing (Kaplan and Kaplan, 1989; Gaekwad et al., 2022), reflecting broader accounts of humans’ innate affinity with living systems (Wilson, 1984). Creative participation is linked to reduced stress, stronger social bonds, and meaning-making (Stuckey and Nobel, 2010; Fancourt and Finn, 2019). Awe and wonder—often elicited through art or nature—are also associated with wellbeing and prosocial behaviors (Bai et al., 2017; Monroy and Keltner, 2023). Yet these domains are often studied separately. Less is known about how immersive practices that combine nature, technology, and co-creation support resilience and identity reconstruction after medical trauma, especially when voice and communication are central.

We addressed this gap through a collaboration of poetic co-creation, group singing, advanced sound capture, and infrared imaging in performance and installation. Nature was not merely background or metaphor. The Hibakujumoku acted as living stimuli, with their recorded vibrations and images woven into the work. In this context, the trees’ endurance became a shared point of reference for those negotiating voice loss and medical intervention.

A participatory, arts-based approach was adopted to access embodied, relational, and affective dimensions of experience that are often difficult to capture through conventional methods. These methods include clinical or interview-only approaches. Co-creation foregrounds lived knowledge, shared authorship, and sensory engagement.

It also offers routes into experience through sound, rhythm, imagery, performance, and dialog. This is particularly relevant to post-laryngectomy contexts where identity and communication are deeply entangled and not always easily articulated in speech.

In line with previous findings (Reagon et al., 2017; Warran et al., 2019; Baker et al., 2019), this paper examines how an immersive art–science collaboration shaped experiences of emotional regulation, identity reconstruction, social connection, and meaning-making, more specifically through the pairing of human and ecological survivorship. The primary research questions guiding this study were: (1) In what ways does engaging with both human and ecological survivor narratives influence participants’ emotional regulation and identity reconstruction following laryngectomy? (2) How does the co-creative process involving nature, technology, and voice affect social connection and the reconstruction of meaning for participants? The Discussion relates these themes directly to arts-in-health, nature-based wellbeing, co-creation, and post-traumatic growth.

## Methodology and methods

### Methodological orientation

This study used a qualitative, arts-based participatory design and reflexive thematic analysis (Braun and Clarke, 2006, 2019). The work followed an interpretivist approach, in which meaning is co-constructed through embodied, relational, and creative practice rather than viewed as an objective property of data. Co-creation was central to both artistic and research design; participants were collaborators whose lived experience and creative contributions shaped the process and outcomes.

Arts-based methods were chosen because post-laryngectomy experience—especially regarding voice, identity, and communication—is deeply embodied and not always suited to traditional interviews or clinical approaches. The study, therefore, prioritised sensory, performative, and relational inquiry.

### Researcher positionality and reflexivity

The research team comprised two researchers with complementary roles. The first author, a clinician–artist, designed and facilitated the workshops and contributed to the analysis. This role supported close engagement with participants’ vocal practice and rehabilitation while requiring attention to power, influence, and interpretive framing. We included an independent psychosocial researcher, who had no clinical relationship with participants or involvement in facilitation. Their role was to provide analytic distance and critical dialog.

Other contributors—composers, musicians, a poet and visual artist—were essential to creation but not part of the research team or analysis. Their reflections are included as data.

Reflexivity was supported by analytic memos and regular researcher discussions. The team considered how facilitation, disciplinary background, and artistic aims may have shaped what was voiced and interpreted. In reflexive thematic analysis, subjectivity was used as an analytic resource rather than seen as a limitation.

### Participants

Ten adults who had total laryngectomy and radiotherapy after head and neck cancer participated. Recruited via Shout at Cancer, ages ranged from 42 to 73; three were female, and seven were male (Table 1).

**Table 1:** Participant characteristics, including age range, gender, time since total laryngectomy, and primary communication method (*n* = 10).

Participant ID	Age range	Gender	Time after surgery (years)	Primary communication method
P1	40–49	F	2	Tracheosophageal speech
P2	70–79	M	25	Tracheosophageal speech
P3	50–59	F	14	Tracheosophageal speech
P4	70–79	M	2	Tracheosophageal speech
P5	40–49	M	5	Tracheosophageal speech
P6	60–69	M	13	Tracheosophageal speech
P7	70–79	M	11	Electrolarynx
P8	60–69	F	13	Tracheosophageal speech
P9	60–69	M	16	Tracheosophageal speech
P10	60–69	M	9	Tracheosophageal speech

### Workshop design and procedure

Six workshops took place over 10 weeks. Each session combined group singing, creative writing, and reflection. Participants co-created poetic and vocal work about voice loss, rehabilitation, resilience, and identity change.

Workshops were led by the clinician–artist researcher, with input from the poet and the other artists. Collaborators shaped the creative space, but research design, data collection, and analysis stayed with the research team.

Participants learned about the histories and recordings of the Hibakujumoku. These stories were used as shared stimuli rather than as metaphors. Participants explored parallels between ecological endurance and their recovery through creative responses.

### Materials and technologies

The internal vibrations and inaudible sounds of the Hibakujumoku were captured with contact microphones, accelerometers, and hydrophones. Recordings occurred in Hiroshima and Nagasaki on 6 and 9 August 2020, marking the 75th anniversary of the atomic bombings, after obtaining approval from local authorities and tree custodians.

**Figure 1:**
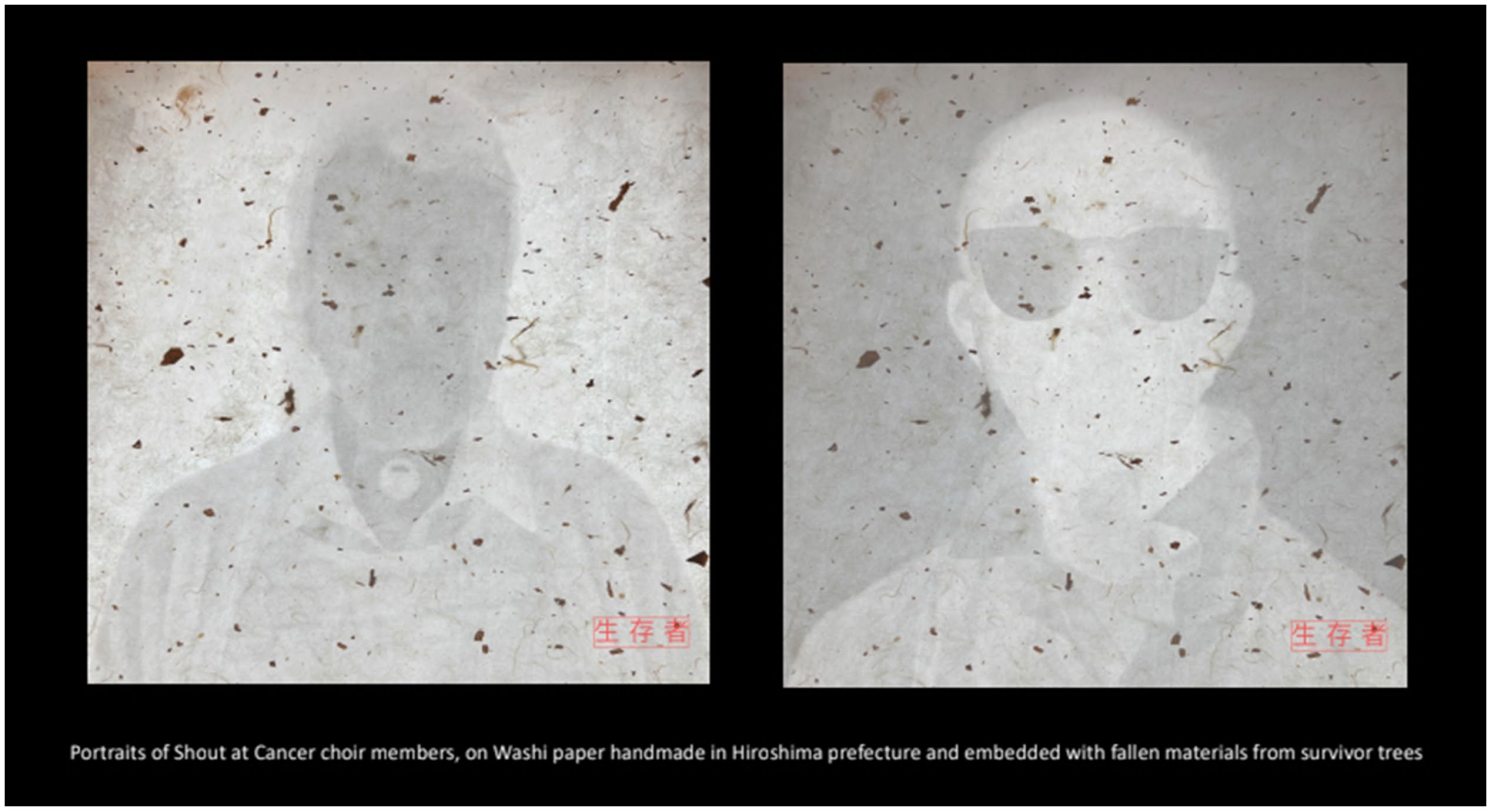
Portraits of Shout at Cancer members printed on handmade Washi paper from Hiroshima Prefecture, embedded with fallen material from Hibakujumoku (survivor trees); thermal imaging technology. Image credit: Philip Clemo. Reprinted with permission from Philip Clemo.

**Figure 2:**
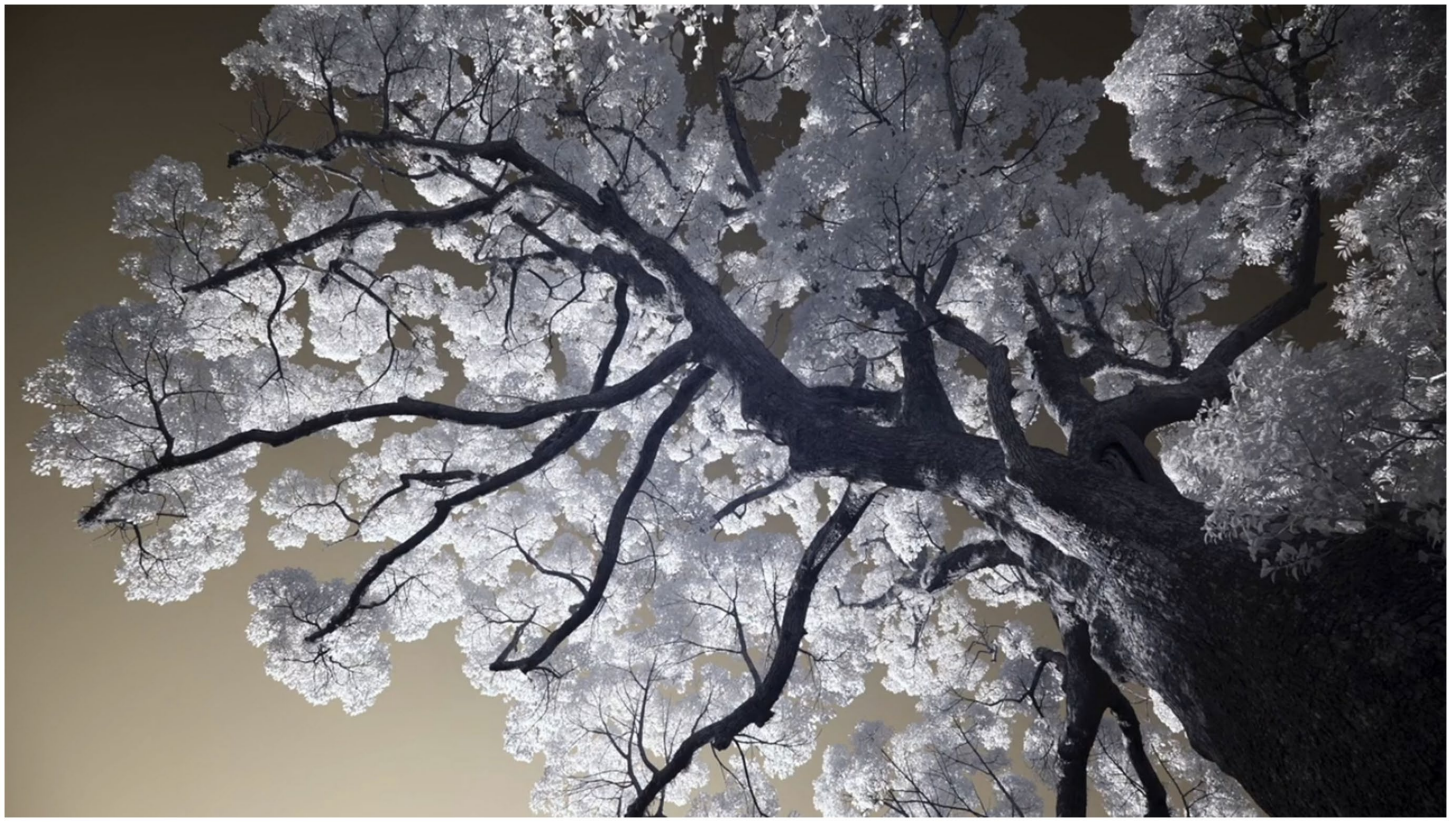
Camphor tree 5, Fuchi Shrine, Nagasaki; infrared imaging technology. Image credit: Philip Clemo and Colin Gray. Reprinted with permission from Philip Clemo.

**Figure 3:**
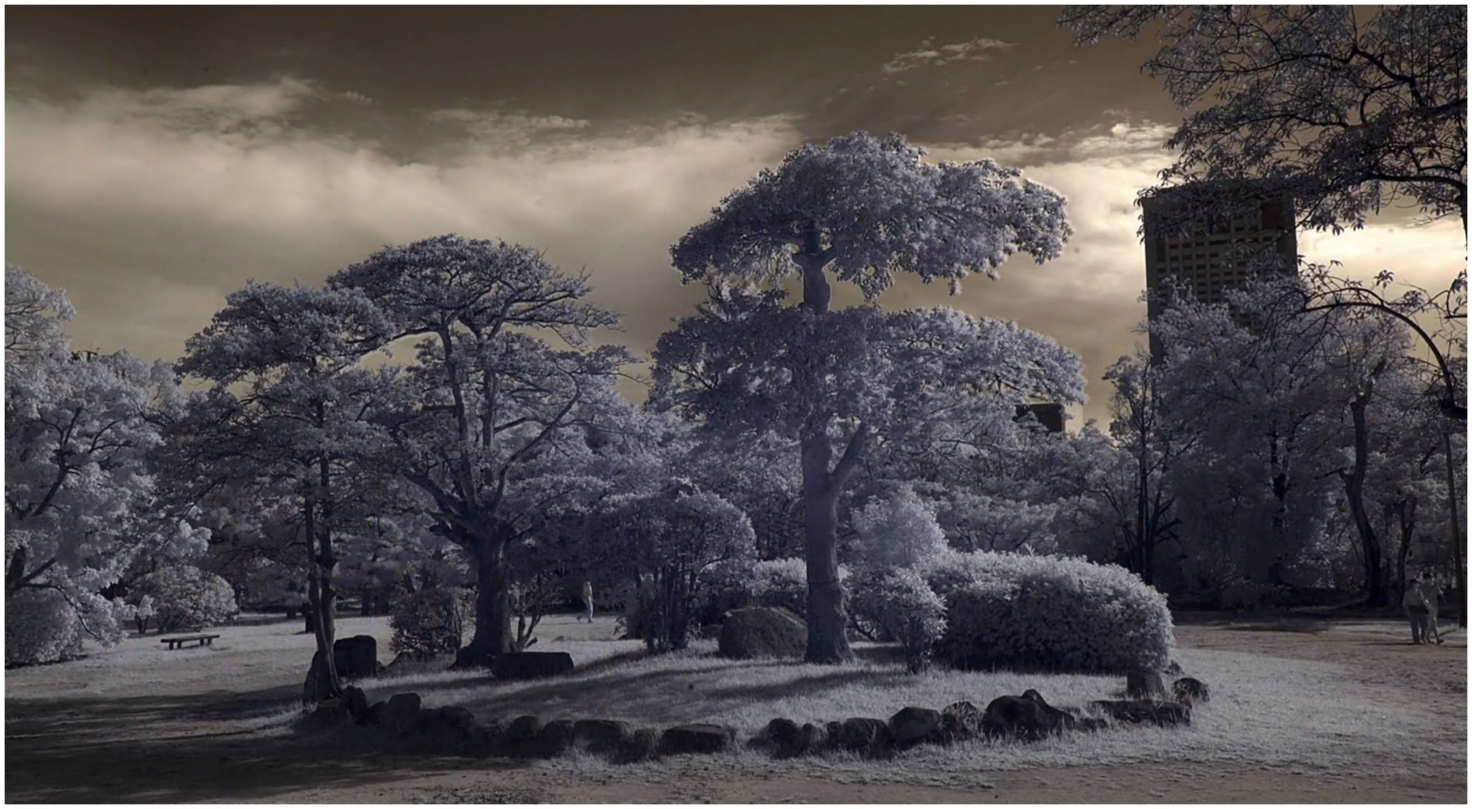
Three Kurogane holly trees, Hiroshima Castle Grounds, Hiroshima; merged infrared and thermal imaging. Image credit: Philip Clemo and Colin Gray. Reprinted with permission from Philip Clemo.

Thermal and infrared imaging visualized physiological and energetic processes invisible to the naked eye (Figures 1–3).

Participants’ vocal sounds were recorded during workshops. These recordings were combined with tree sounds to create hybrid compositions by collaborating composers and sound artists. Later, visual “voice sculptures” were generated using digital systems developed by Harry Yeff (Reeps One) and Trung Bao, translating vocal sound and intensity into sculptural visual forms (Voice Gems: 1,000 year archive). These artifacts functioned as creative outputs and stimuli rather than analytic units.

### Data sources

The dataset comprised:

- notes and audio recordings of workshops and rehearsals
- creative texts (poems, lyrics, written reflections)
- audio-visual documentation of performances and installations
- researchers’ field notes and reflexive memos

Informal audience feedback was recorded in field notes to contextualise reception and was not subjected to formal thematic analysis.

### Data analysis

Data were analysed using reflexive thematic analysis (Braun and Clarke, 2006, 2019). Analysis was conducted by the two members of the research team.

Both researchers immersed themselves through repeated reading, listening, and viewing. Initial codes were generated inductively, guided by the study focus (voice, survivorship, ecological material, and co-creation) without imposing a predefined framework. Codes were iteratively organised into themes through analytic discussion, with attention to shared patterns of meaning.

Given the facilitator’s dual role, reflexive practices were embedded throughout. Assumptions, emotional responses, and facilitation dynamics were documented and examined. Themes were reviewed against the dataset for coherence and distinctiveness, then refined and named.

Credibility was supported through triangulation, which involved systematically comparing findings across multiple data types, including workshop notes, audio recordings, creative texts, and researcher field notes. In addition, participants reflected on workshop outputs to provide further perspectives on emerging interpretations, with resonance rather than verification prioritized as the marker of analytic rigor. Coding consensus and inter-rater reliability were not sought, consistent with a reflexive approach (Braun and Clarke, 2006, 2019).

### Ethical and cultural considerations

Engagement with the Hibakujumoku was conducted respectfully and in consultation with relevant custodians, including The Green Legacy Hiroshima, recognising the historical and cultural sensitivity of Hiroshima and Nagasaki.

Safeguarding included flexible participation, optional engagement levels, post-session debriefing, and access to clinical support. In line with performance-care ethics in singing voice rehabilitation, particular attention was paid to addressing power dynamics and hierarchical relationships between clinician–researchers and participants, including participant agency over modes of participation and representation (Brown, 2025). All participants provided written informed consent for participation and for the use of de-identified extracts in publications and performances.

The study complied with the Declaration of Helsinki. Further ethical approval was not required under UK guidelines, as confirmed by the MRC/NHS HRA decision tool (also, see “Ethics” statement, below).

## Results

This section reports findings from a reflexive thematic analysis of workshop discussions and transcripts, participants’ reflections, performance-related feedback, and researchers’ field notes. Creative texts (poems/lyrics) were treated as data only where they functioned as reflective accounts (e.g., identity, coping, social experience) rather than as aesthetic artifacts. Written reflections and field notes from collaborating composers, the writer, and the visual artist were included where they formed part of the analytic dataset. Artistic outputs (compositions, performances, installations) are reported descriptively as contextual outcomes and were not themselves analysed thematically.

Three themes were developed: Parallel Survivorship, Reclaimed Agency, and Collective Embodiment. Several subthemes—Re-voicing Identity, Humor, Dignity, and Everyday Coping, and Rediscovering the Body and Sensory Adaptation—were coded across multiple themes. Extracts are attributed by role and, where available, by participant ID.

### Theme 1: parallel survivorship

Parallel survivorship describes how survivorship was articulated through the relationship between the Hibakujumoku and participants’ experience after cancer treatment, including radiotherapy. Across accounts, the trees were repeatedly positioned as enduring and still communicative.

#### Participants: survivorship as a shared condition; silence as vitality

Participants linked the trees’ endurance to their own survival:

- “These trees survived an atomic explosion and still prosper — their resilience inspired us all.” (P5)
- “The trees and we share the same radiation, but both of us are still here, still growing.” (P3)
- “Laryngectomee or not, most of us will experience illness or trauma in our lives and need such treelike resilience to endure.” (P6)

Participants also described surprise at the tree recordings and a shift in assumptions about silence:

- “I was flabbergasted that trees made such sounds… I would have considered that person demented who told me trees could talk!” (P6)

#### Composers: hearing the trees through hearing the choir

Composers described how familiarity with the choir shaped how they approached the tree recordings:

- “It became easier to understand the trees after I better understood the voices of the choir.” (Composer)
- “At first the weight of the project was overwhelming… Once I immersed myself in the minute detail of the tree sounds, I was able to hear what they were saying musically.” (Composer)

#### Visual artist: trees as witnesses; parallel re-voicing

The visual artist framed the trees as historical witnesses and linked “revealing” hidden vitality to the choir’s recovery:

- “A Nagasaki survivor told me that soon these trees will be the only living witnesses of the bombs.” (Visual artist)
- “We uncovered the trees’ hidden voices, just as … the choir members rediscovered theirs. Both were silent, and now they sing together in harmony.” (Visual artist)

Reflecting on the project as a whole, one participant summarised this sense of shared endurance: “The sounds of the trees echoed our own stories of survival… like them, we have endured destruction and found new ways to grow.”

### Theme 2: reclaimed agency

Reclaimed agency describes how participants spoke about confidence, control, and public presence developing through co-creation and performance. Agency was described both in everyday interactions and on stage.

#### Participants: performance as reclamation; audience as a partner in listening

Participants described performance as a way of reclaiming voice:

- “For a ‘voiceless’ person to convey emotion through altered speech was an act of reclamation.” (P4)

They also described shifts in perceived power relations with audiences:

- “We’re in control — they are listening!” (P3)
- “You have to train the audience — they do more effort; their listening improves.” (P2)

Participants positioned the work as advocacy and support for others:

- “It’s all about helping others regain confidence.” (P8)
- “So proud to be part of the choir — to make a difference for anyone who has to go through the same thing.” (P7)

#### Cross-cutting subtheme: re-voicing identity

Identity was described as an ongoing negotiation, with ambivalence alongside acceptance:

- “I thought the operation would not define me — but it did, and I fight it every day.” (P10)
- “After enjoying my experience with the choir, I love the new sound of my voice.” (P5)
- “I, as a ‘voiceless’ laryngectomee, could show that I had varied intonation and volume…” (P9)

#### Cross-cutting subtheme: humor, dignity, and everyday coping

Humor appeared as a practical resource for handling stigma and maintaining control:

- “Amazon driver tells me that I sound rough: ‘I do not care — I got my package.’” (P10)

#### Writer: authorship as service; listening as method

The writer described shifts away from self-display and toward service and attentive listening:

- “It gave me a way to use my performance and writing skills for the good of others, with no lens put on myself.” (Writer)
- “Listening and learning about the people in the program was the most meaningful part.” (Writer)
- “You come face to face with the fact that the voice is a precious gift, as is the choice of silence.” (Writer)

### Theme 3: collective embodiment

Collective Embodiment describes experiences of shared synchrony, mutual attention, and “co-sounding” during group singing and performance. Voice was often described as distributed across bodies, rhythms, and sound forms.

#### Participants: uplift, belonging, and sound beyond speech

Participants described shared lift and confidence:

- “Singing together brought elation and confidence — a lifting of spirit.” (P4)

They also broadened what counted as communication:

- “Emergence proved that all sound can be a form of communication, whether it be a hum or click or tut.” (P6)

Participants described the integration of choir and tree sounds as coherent:

- “To hear our voices merge with the sound of the trees was enlightening and inspiring… when combined it all fell into place.” (P1)

#### Composers: the project as a living, shared organism

Composers described collective authorship and the evolving character of the work:

- “It was like nothing else I’d ever worked on… The whole project itself feels like it’s living, growing, constantly branching outwards in unexpected tangents.” (Composer)
- “The friendships and connections made with the collaborators have had a profound impact on my personal and artistic life.” (Composer)

#### Visual artist: making vitality visible

The visual artist described imaging as a way of rendering vitality perceptible:

- “I wanted to capture something of their powerful life-force, so I used infrared and military-grade thermal cameras to reveal the trees beyond the ordinary.” (Visual artist)

#### Cross-cutting subtheme: rediscovering the body and sensory adaptation

Listening and changes in pacing were described as part of adaptation:

- “Listening has a healing aspect; I can slow sounds down.” (P4)

### Descriptive outcomes of the creative process

Across six workshops, participants collaborated with poet Bruce Sherfield to co-create poems and lyrics, which were later integrated into interdisciplinary compositions by Christian Drew, Reeps One (Harry Yeff), and Teddy Riley. Tree recordings (contact microphones, accelerometers, hydrophones) and thermal/infrared imaging were incorporated into performance and installation contexts (Philip Clemo). These outputs provide context for the experiences reported above but were not treated as thematic data.

### Analytic transparency

Supplementary material supports auditability. Supplement 3 outlines workshop structures and activities. Supplement 4 maps data sources to themes and subthemes. Supplements 5–8 provide the analytic record for participant and collaborator perspectives (codes, example extracts, and analytic notes).

## Discussion

### Unique contribution to the literature

This study aims to contribute to the literature at the intersection of arts-in-health, ecological metaphor, and participatory recovery after medical trauma (Fancourt and Finn, 2019; Daykin, 2012). Unlike previous research, which has often examined human and ecological survivorship separately or has focused primarily on either clinical or symbolic dimensions (Kaplan and Kaplan, 1989; Stuckey and Nobel, 2010), this work demonstrates how immersive, co-creative art–science practice can shape experiences of recovery following laryngectomy. By integrating technologically captured tree vitality and human vocal rehabilitation, the study offers a new model for how nature, technology, and creative participation can be intertwined to foster emotional regulation, identity reconstruction, and social connectedness (Baker et al., 2019; Warran et al., 2019).

Instead of positioning ecological material as mere metaphor or atmospherics, the findings show that actively engaging with non-human survivor narratives and integrating their sensory traces within group artistic practice enables novel forms of meaning-making and relational wellbeing (Raw et al., 2012; Weeseman et al., 2023). This interdisciplinary approach extends current scholarship by highlighting the reciprocal dynamics of co-creation among participants and collaborators, and by reframing silence and altered voice within a broader ecology of survivorship. As such, the study provides an innovative framework for future research on arts-based interventions and offers a foundation for exploring the mutual benefits of art–science collaborations for both clinical and public health contexts.

This study examined how an immersive, co-creative art–science process shaped experiences of recovery and communication among people living after laryngectomy in dialog with the Hibakujumoku. Across the dataset, three linked patterns were evident: recognising survivorship across human and ecological domains (*Parallel Survivorship*), reclaiming public presence through creative authorship (*Reclaimed Agency*), and experiencing voice as shared, embodied synchrony rather than individual sound production (*Collective Embodiment*). This section interprets these themes in relation to work on arts-in-health, nature-based wellbeing, and co-creation.

### Parallel survivorship

Participants described the Hibakujumoku as more than a symbolic backdrop. Tree sound and image offered a concrete encounter that supported reflection on endurance, injury, and continued life. This is consistent with literature on contact with nature and restoration of attention (Kaplan and Kaplan, 1989), as well as broader accounts of humans’ innate affinity with living systems (Wilson, 1984), and work linking awe to expanded perspective, orientation to relationships, and social connection (Piff et al., 2015; Bai et al., 2017; Monroy and Keltner, 2023). In this project, awe was tied to a specific shift in how “silence” was understood—less as absence and more as hidden vitality—providing a basis for later co-creative work.

The findings also extend arts-and-health accounts by demonstrating that ecological material can take an active role in meaning-making rather than merely providing a calming setting. For example, recordings of the Hibakujumoku trees’ internal vibrations, captured through contact microphones and accelerometers, were played during workshops and prompted participants to reflect on the persistence of life beneath apparent destruction. Similarly, the thermal and infrared images, which revealed physiological processes within the trees invisible to the naked eye, provided a visual metaphor and tangible evidence of hidden vitality. These multimodal ecological data enabled participants to conceptualize survivorship as an ongoing process beneath visible damage, a perspective that was echoed across participant and collaborator accounts.

Interpreted through meaning-centered and coping-oriented frameworks (Breitbart et al., 2010), this reframing of survivorship can be understood as a process of narrative coherence rather than therapeutic resolution. Aligning personal experiences of recovery with the natural world’s endurance offered participants a way to situate their loss. This reframing placed their survival within a broader, ongoing story. In this sense, the survivor trees functioned less as imposed symbols and more as experiential reference points through which participants articulated perspective, continuity, and acceptance.

### Reclaimed agency

Participants did not describe voice recovery only in terms of clinical function; they described authorship, performance, and the terms on which they were heard. This aligns with research on participatory arts and group singing as routes to confidence, identity reconstruction, and social reintegration (Warran et al., 2019; Baker et al., 2019; Weinstein et al., 2016). Agency was also described as relational: participants spoke about “training” audiences to listen. This shifts communicative labor away from the speaker alone and onto the interaction itself, while remaining grounded in everyday practice.

Co-creation was central to this theme. Participants framed themselves as makers rather than recipients, consistent with arguments that shared authorship can restore agency after illness-related disruption (Reagon et al., 2017; Baker et al., 2019). Humor further underscored agency: it served to manage stigma, protect dignity, and control the narrative in public encounters. This emphasis on shared authorship and relational listening resonates with narrative accounts of inclusion through collective song, where participants describe belonging as emerging through being heard *with* others rather than speaking alone (Brown and Ranganathan, 2025).

### Collective embodiment and “voice beyond voice”

Collective Embodiment aligns with established findings that group singing supports bonding and synchrony (Weinstein et al., 2016; Hallam and Himonides, 2022). Here, participants repeatedly described communication in small sound units (hum, click, tut), reflecting a widened definition of voice grounded in rhythm, breath, and shared attention.

A distinctive feature of this project was that technological mediation was described as intensifying shared experience rather than distancing it. Tree vibrations and thermal/infrared imagery offered additional channels for participants to perceive themselves as part of a wider “sounding ecology.” In this sense, voice was framed as relational energy—vibration, timing, and attention—rather than solely as vocal anatomy.

### Collaborator reflections as part of the dataset

Collaborator extracts are reported here as data in their own right rather than as commentary on participants. They show how interdisciplinary co-creation shaped the perspectives and practices of those contributing to the work, supporting accounts of co-creation as reciprocal rather than unidirectional (Raw et al., 2012; Daykin, 2012; Weeseman et al., 2023). Across collaborators’ reflections, listening, service, and shared authorship were repeated concerns, suggesting that the effects of the work were distributed across the collaboration rather than located only in participant “benefit.”

### Relation to prior research

The participants’ reflections vividly illustrate well-documented health and well-being benefits of arts engagement, particularly within the domains of emotional expression, identity reconstruction, social connection, and meaning-making.

#### Emotional regulation and psychological wellbeing

Hearing the “voices” of the survivor trees evoked awe and wonder—emotions associated with reduced stress, enhanced mood, and increased sense of connectedness (Stellar et al., 2015; Piff et al., 2015; Bai et al., 2017; Monroy and Keltner, 2023). Listening to their inner vibrations created an embodied sense of peace and connectedness. For people recovering from medical trauma, this nature-based resonance offered an alternative form of mind–body regulation—lowering anxiety, fostering gratitude, and enhancing emotional balance.

Simply looking at the beautiful images and videos of the trees evoked calm and reflection; participants’ responses can be interpreted through restoration of attention (Kaplan and Kaplan, 1989, pp. 170–190) and the Biophilia Hypothesis (Wilson, 1984, pp. 1–12; Gaekwad et al., 2022). These frameworks propose that exposure to the aesthetic and mysterious qualities of nature restores directed attention, alleviates cognitive fatigue, and enhances emotional and spiritual well-being. “I was flabbergasted that trees made such sounds”—reflects this restorative shift from inner tension to outward fascination, inviting calm attention and renewed mental clarity.

The act of singing and performing allowed participants to express vulnerability safely, transforming trauma into creativity. This aligns with evidence showing that arts participation supports emotional regulation, reduces anxiety and depression, and builds resilience through cathartic expression and shared joy (Fancourt et al., 2019; Coulton et al., 2015; Hallam and Himonides, 2022, pp. 413–478). Singing with the “voices” of trees blurred boundaries between human and non-human communication, nurturing a sense of ecological belonging. Participants described it as a “conversation between human and nature,” echoing evidence that nature contact fosters transcendence, gratitude, and empathy (Gaekwad et al., 2022).

#### Identity, confidence, and agency

For individuals living with voice loss after laryngectomy, performing poetry and song represented reclamation of identity and reassertion of agency. Discovering that communication “transcends the conventional voice” supported self-efficacy and post-traumatic growth—key psychological outcomes of participatory arts, especially in rehabilitation contexts (Baker et al., 2019; Skewes McFerran et al., 2020; Warran et al., 2019; Bickford et al., 2018).

#### Social connection and collective healing

Participation in the choir created an ego-less, supportive community where vulnerability was shared and valued. Research in community arts and group singing consistently links such participation with enhanced social bonding, reduced loneliness, and a greater sense of belonging —all predictors of improved health outcomes (Warran et al., 2019;Weinstein et al., 2016; Hallam and Himonides, 2022, pp. 551–559). Recent narrative work on young people’s experiences of inclusion through shared song similarly highlights how collective music-making fosters belonging and psychosocial safety (Brown and Ranganathan, 2025), echoing the sense of “ego-less” community described by participants in the present study.

#### Meaning, purpose, and existential wellbeing

The metaphor of the ‘survivor trees’ offered participants a salient symbolic framework through which to interpret personal resilience and renewal, thereby enhancing spiritual wellbeing and facilitating adaptive reappraisal of illness experiences. This process of aligning individual narratives of recovery with the observable phenomenon of natural regeneration enabled participants to construct coherence, derive meaning, and attain psychological comfort and perspective, all of which contribute substantively to long-term mental health outcomes (Breitbart et al., 2010; Folkman, 2008). This symbolic identification is foundational to the concept of ecopsychological healing, which posits that meaningful connections with resilient aspects of the natural environment support processes of meaning-making, acceptance of adversity, and the cultivation of renewed hope during psychological recovery.

### Relation to the therapeutic potential of co-creation

#### Shared ownership and agency

Co-creation places participants not as subjects but as collaborators. In this project, individuals who had lost their natural voices became active creators—shaping lyrics, poems, and performances alongside professional artists. This restored a sense of agency and authorship often diminished after medical trauma (Baker et al., 2019; Warran et al., 2019). Co-performing exemplified how creative contribution reclaims identity: speaking and shaping meaning rather than being defined by loss (Baker et al., 2019; Fancourt et al., 2019; Reagon et al., 2017; Warran et al., 2019).

#### Empathy and mutual recognition

The process built a reciprocal dialog between artists, scientists, and participants. Each listened and responded to the other—human voices to tree vibrations, composers to lived experiences, performers to audience. This dynamic exchange is central to relational aesthetics and therapeutic co-creation, fostering empathy, equality, and mutual respect (Batalden et al., 2016; Weeseman et al., 2023). Participants described it as an “ego-less environment,” echoing research showing that shared creative purpose reduces isolation and enhances social cohesion.

#### Transforming vulnerability into expression

Co-creation allowed participants to turn perceived limitations into creative resources. The altered, mechanical voices of the choir became instruments of beauty; their vulnerability became art. This transformation parallels therapeutic models like arts-based narrative therapy, where expression reframes trauma into growth and meaning, supporting post-traumatic resilience and emotional integration (Gallagher et al., 2018; Stuckey and Nobel, 2010).

#### Embodied and collective healing

Singing together—and with the trees—engaged participants in a communal act of embodiment. Co-creating sound, rhythm, and breath synchronised bodies and emotions, promoting physiological regulation, confidence, and joy. Such shared artistic flow states are known to release endorphins and oxytocin, enhancing mood and social bonding (Keeler et al., 2015; Weinstein et al., 2016).

#### Meaning-making and continuity of self

The act of co-creating across human and natural voices bridged science, art, and spirituality. It provided participants with a new narrative continuity: they were no longer “patients” but contributors to a living artwork that symbolised renewal. This aligns with research on the therapeutic role of participatory arts in fostering meaning, coherence, and existential well-being after illness (Baker et al., 2019; Bickford et al., 2018; Stuckey and Nobel, 2010; Weinstein et al., 2016).

Through co-creation, participants moved from isolation to collaboration, from silence to shared authorship. The process itself—listening, shaping, performing—became therapeutic, enabling emotional release, social connection, and the re-establishment of identity. In blending art, science, and lived experience, the project demonstrates that co-creation is not only creative but restorative: it heals through participation, empathy, and the collective rediscovery of voice.

#### Integrating the artists’ perspective: the mutual benefits of co-creation

While the project was designed to support laryngectomy participants in reclaiming voice and confidence, the artists, too, underwent a process of transformation. Their reflections reveal that co-creation functioned as a two-way therapeutic exchange (Raw et al., 2012; Weeseman et al., 2023)—an encounter that reshaped artistic practice, deepened empathy, and expanded their sense of purpose.

#### Empathic growth and perspective shift

Through intimate collaboration, artists experienced a profound shift from artistic control to active listening and shared authorship (Daykin, 2012; Weeseman et al., 2023). The composer described learning to “understand the trees’ voices through the choir’s voices,” while the writer and visual artist both emphasised that the process redefined their understanding of what voice, silence, and communication mean. This mirrors research showing that co-creative arts practice enhances empathy, relational awareness, and reflective capacity in artists, fostering a more human-centered approach to creativity (Daykin, 2012; Raw et al., 2012; Weeseman et al., 2023).

#### Emotional and existential enrichment

Each artist described the experience as personally transformative. Working alongside people who had lost and regained their voices reframed their own assumptions about expression, fragility, and resilience. The visual artist found the project “spiritual,” a meditation on endurance and renewal. The composer spoke of balancing “scientific precision with emotional authenticity.” Such encounters exemplify the restorative emotional impact of socially engaged art-making—often cited as improving artists’ sense of meaning, gratitude, and psychological wellbeing (Daykin, 2012; Raw et al., 2012; Stuckey and Nobel, 2010; Weeseman et al., 2023).

#### Purpose beyond performance

The writer articulated the observation that this project shifted their practice from performance toward service and communion—using creative skill “not for recognition but to help others find voice.” Similarly, the composer described composition as “weaving together” rather than leading—reflecting the therapeutic ethos of humility, reciprocity, and shared purpose. Participating in a project of collective resilience gave each artist a renewed sense of professional and moral clarity: that art can heal, educate, and connect (Daykin, 2012; Fancourt and Finn, 2019; Raw et al., 2012; Stuckey and Nobel, 2010; Weeseman et al., 2023).

#### Creative renewal and expanded practice

The unpredictable, improvisatory nature of the collaboration—combining poetry, music, and tree vibrations—required trust and openness to “happy accidents.” This process reinvigorated the artists’ creativity, pushing them beyond familiar techniques and into cross-disciplinary innovation. Such experiences align with research suggesting that co-creation stimulates cognitive flexibility, flow, and long-term creative renewal, enhancing both professional satisfaction and artistic evolution (Fancourt and Finn, 2019; Weeseman et al., 2023).

The therapeutic value of the project extended in both directions. For participants, it nurtured healing, confidence, and connection; for artists, it provided empathic growth, creative renewal, and existential grounding. Together they formed a community where art became a shared act of care—a living example of how co-creation not only supports recovery but also sustains the wellbeing and purpose of those who facilitate it.

### Limitations

This was a qualitative, context-specific study with a small sample drawn from an established choir. The findings are therefore highly contextualised and not argued to be statistically generalisable. Participants may also have been more willing than some laryngectomy survivors to engage in group music-making and public performance, which may have shaped the reported experiences presented in this study. The facilitator’s dual role as clinician–artist could have influenced what was voiced in the workshops; this was addressed through ongoing reflexive memoing and analytic dialog with an independent researcher. These points are believed not to be detrimental to the study; they are offered as points of clarification about the parameters and conditions under which the findings were identified.

### Future directions

Future work could examine how similar co-creative designs function in other clinical and cultural settings, including with people earlier in recovery or with less access to peer support. Mixed-methods and longitudinal approaches may be useful where they answer specific questions (e.g., accessibility, durability of reported changes, and likely mechanisms), rather than being framed as “validation” of qualitative results. Further studies could also compare different ecological inputs (sound, image, site-based encounter) and test how different forms of technological mediation shape meaning-making, listening practices, and participation. Such work could also draw on emerging and historical models of vocal tract representation and simulation, including developmental accounts of the Vocal Tract Organ, to explore how alternative vocal systems and technological proxies might further expand concepts of voice, embodiment, and communicative agency in rehabilitation contexts (Howard, 2025).

## Conclusion

This study suggests that an immersive art–science collaboration can support recovery narratives that move beyond deficit accounts of voice loss. Participants described voice as vibration and presence, communication as shared work with listeners, and survivorship as a form of endurance articulated across human and ecological domains. By placing people post-laryngectomy in dialog with the Hibakujumoku through listening, co-creation, and performance, the project created conditions in which silence could be approached as hidden vitality and altered voice as public authorship. These findings indicate that carefully designed, ethically attentive co-creative work may open forms of meaning, connection, and collective resilience that sit alongside—rather than replace—clinical rehabilitation.

## Data Availability

De-identified data supporting the conclusions of this article will be made available by the authors, without undue reservation.

## References

[ref1] Bai Y. Maruskin L. A. Chen S. Gordon A. M. Stellar J. E. McNeil G. D. . (2017). Awe, the diminished self, and collective engagement: universals and cultural variations in the small self. J. Pers. Soc. Psychol. 113, 185–209. doi: 10.1037/pspa0000087, 28481617

[ref2] Baker F. A. Tamplin J. Rickard N. Ponsford J. New P. W. Lee Y.-E. C. (2019). A therapeutic songwriting intervention to promote reconstruction of self-concept and enhance well-being following brain or spinal cord injury: pilot randomized controlled trial. Clin. Rehabil. 33, 1045–1055. doi: 10.1177/0269215519831417, 30791702

[ref3] Batalden M. Batalden P. Margolis P. Seid M. Armstrong G. Opipari-Arrigan L. . (2016). Coproduction of healthcare service. BMJ Quality & Safety 25, 509–517. doi: 10.1136/bmjqs-2015-004315, 26376674 PMC4941163

[ref4] Bickford J. M. Coveney J. Baker J. Hersh D. (2018). Self-expression and identity after total laryngectomy: implications for support. Psycho-Oncolgy 27, 2638–2644. doi: 10.1002/pon.4818, 29927018

[ref5] Braun V. Clarke V. (2006). Using thematic analysis in psychology. Qual. Res. Psychol. 3, 77–101. doi: 10.1191/1478088706qp063oa

[ref6] Braun V. Clarke V. (2019). Reflecting on reflexive thematic analysis. Qual. Res. Sport Exerc. Health 11, 589–597. doi: 10.1080/2159676X.2019.1628806

[ref7] Breitbart W. Poppito S. R. Rosenfeld B. Vickers A. J. Li Y. Abbey J. . (2010). Meaning-centered psychotherapy for patients with advanced cancer: a pilot randomized controlled trial. Psycho-Oncology 19, 21–28. doi: 10.1002/pon.1556, 19274623 PMC3648880

[ref8] Brown J. (2025). The ethics of performance care: a pragmatic feminist analysis of policy for singing voice rehabilitation. J. Eval. Clin. Pract. 31:e14107. doi: 10.1111/jep.14107, 39347605 PMC11938413

[ref9] Brown J. Ranganathan S. (2025). ‘Sharing my voice’: a young person’s story of inclusion and belonging through shared song. Music Arts Action 9, 26–45. Available at: https://musicandartsinaction.net/index.php/maia/article/view/271/sharingmyvoice

[ref10] Coulton S. Clift S. Skingley A. Rodriguez J. (2015). Effectiveness and cost-effectiveness of community singing on mental health-related quality of life of older people: randomised controlled trial. Br. J. Psychiatry 207, 250–255. doi: 10.1192/bjp.bp.113.129908, 26089304

[ref11] Daykin N. (2012). “Developing social models for research and practice in music, arts and health: a case study of research in a mental health setting” in Music, health, and wellbeing (Oxford, UK: Oxford University Press), 65–75.

[ref12] Fancourt D. Finn S., 2019. What is the evidence on the role of the arts in improving health and well-being? A scoping review. Copenhagen: WHO Regional Office for Europe 32091683

[ref13] Fancourt D. Warran K. Finn S. Wiseman T. (2019). Psychosocial singing interventions for the mental health and well-being of older people: a systematic review and meta-analysis. Med. Humanit. 9, 272–285. doi: 10.1136/bmjopen-2018-026995, 31401592 PMC6701813

[ref14] Folkman S. (2008). The case for positive emotions in the stress process. Anxiety, Stress & Coping 21, 3–14. doi: 10.1080/10615800701740457, 18027121

[ref15] Gaekwad J. S. Sal Moslehian A. Roös P. B. Walker A. (2022). A meta-analysis of emotional evidence for the biophilia hypothesis and implications for biophilic design. Front. Psychol. 13:750245. doi: 10.3389/fpsyg.2022.750245, 35693493 PMC9186521

[ref16] Gallagher M. W. Long L. J. Tsai W. Stanton A. L. Lu Q., 2018. The unexpected impact of expressive writing on posttraumatic stress and growth in Chinese American breast cancer survivors. J. Clin. Psychol., 74(10), 1673–1686. 10.1002/jclp.22636, 29727480

[ref17] Hallam S. Himonides E. (2022). The power of music: an exploration of the evidence. Cambridge: Open Book Publishers.

[ref18] Howard D. M. (2025). Developmental history of the vocal tract organ. J. Voice 39, 57–64. doi: 10.1016/j.jvoice.2022.06.025, 35906177

[ref19] Kaplan R. Kaplan S. (1989). The experience of nature: a psychological perspective. Cambridge: Cambridge University Press.

[ref20] Keeler J. R. Roth E. A. Neuser B. L. Spitsbergen J. M. Waters D. J. M. Vianney J.-M. (2015). The neurochemistry and social flow of singing: bonding and oxytocin. Front. Hum. Neurosci. 9:518. doi: 10.3389/fnhum.2015.00518, 26441614 PMC4585277

[ref21] Masten A. S. Best K. M. Garmezy N. (1990). Resilience and development: contributions from the study of children who overcome adversity. Dev. Psychopathol. 2, 425–444. doi: 10.1017/S0954579400005812

[ref22] Monroy M. Keltner D. (2023). Awe as a pathway to mental and physical health. Perspect. Psychol. Sci. 18, 309–320. doi: 10.1177/17456916221094856, 35994778 PMC10018061

[ref23] Piff P. K. Dietze P. Feinberg M. Stancato D. M. Keltner D. (2015). Awe, the small self, and prosocial behavior. J. Pers. Soc. Psychol. 108, 883–899. doi: 10.1037/pspi0000018, 25984788

[ref24] Raw A. Lewis S. Russell A. Macnaughton J. (2012). A hole in the heart: confronting the drive for evidence-based impact in arts and health. Arts & Health 4, 97–108. doi: 10.1080/17533015.2011.619991, 24244217 PMC3827737

[ref25] Reagon C. Gale N. Dow R. Lewis I. van Deursen R. (2017). Choir singing and health status in people affected by cancer. Eur. J. Cancer Care 26:e12568. doi: 10.1111/ecc.12568, 27647701

[ref26] Seery M. D. Holman E. A. Silver R. C. (2010). Whatever does not kill us: cumulative lifetime adversity, vulnerability, and resilience. J. Pers. Soc. Psychol. 99, 1025–1041. doi: 10.1037/a0021344, 20939649

[ref27] Skewes McFerran K. Lai H. I. C. Chang W.-H. Acquaro D. Chin T. C. Stokes H. . (2020). Music, rhythm and trauma: a critical interpretive synthesis of research literature. Front. Psychol. 11:324. doi: 10.3389/fpsyg.2020.00324, 32180753 PMC7059618

[ref28] Stellar J. E. John-Henderson N. Anderson C. L. Gordon A. M. McNeil G. D. Keltner D. (2015). Positive affect and markers of inflammation: discrete positive emotions predict lower levels of inflammatory cytokines. Emotion 15, 129–133. doi: 10.1037/emo0000033, 25603133

[ref29] Stuckey H. L. Nobel J. (2010). The connection between art, healing, and public health: a review of current literature. Am. J. Public Health 100, 254–263. doi: 10.2105/AJPH.2008.156497, 20019311 PMC2804629

[ref30] Warran K. Fancourt D. Wiseman T. (2019). How does the process of group singing impact on people affected by cancer? A grounded theory study. BMJ Open 9:e023261. doi: 10.1136/bmjopen-2018-023261, 30617100 PMC6326295

[ref31] Weeseman Y. Scherer-Rath M. Christophe N. Dörr H. Helmich E. Sprangers M. A. G. . (2023). Co-creative art processes with cancer patients from the artists’ perspective: a qualitative study exploring resonance theory. Support. Care Cancer 31, 287–212. doi: 10.1007/s00520-023-07744-0, 37079143 PMC10119232

[ref32] Weinstein D. Launay J. Pearce E. Dunbar R. I. M. Stewart L. (2016). Singing and social bonding: changes in connectivity and pain threshold as a function of group size. Evol. Hum. Behav. 37, 152–158. doi: 10.1016/j.evolhumbehav.2015.10.002, 27158219 PMC4856205

[ref33] Wilson E. O. (1984). Biophilia. Cambridge, MA: Harvard University Press.

