# Supplementary material for: From silence into song: an art–science collaboration with survivor trees and laryngectomy singers
Source: Front Psychol. 2026 Jan 30;16:1747218. doi: 10.3389/fpsyg.2025.1747218 (PMC12903274; doi:10.3389/fpsyg.2025.1747218)
Supplement: Supplementary file 1 [file Supplementary_file_1.docx]

# **Supplement 1. Project Dissemination (Performances, Installations, and Public Engagement)**

This supplement provides an overview of the dissemination activities associated with the *From Silence into Song* and *Emergence* projects. The outputs were shared through live performances, screenings, installations, outreach broadcasts, and artistic collaborations. These events contributed to public engagement, audience reach, and external creative integration of the project’s sound material.

## **1. Summary of Performances and Screenings**

### **Table S1.1. Public presentations of project outputs**

| **Event / Venue** | **Location** | **Date** | **Format** | **Audience** | **Notes** |
| --- | --- | --- | --- | --- | --- |
| **World Choir Games** | Ghent, Belgium | Oct 2021 | Live streaming | Approx. 4000 | First public presentation of integrated tree–choir sound material. |
| **De Bijloke Concert Hall** | Ghent, Belgium | Oct 2021 | Live performance | Approx 400 | Performed as part of the World Choir Games festival programme. |
| **The Bloomsbury Theatre** | London, UK | Oct 2022 | Live performance + projections | Approx. 250 | Included infrared/thermal tree imagery and survivor-tree soundscore. |
| **Princess Alexandra Hall, Royal Over-Seas League** | London, UK | Apr 2023 | Live performance | Approx. 150 | Featured co-created poems and tree–voice compositions |
| **Lincoln Arts Centre** | Lincoln, UK | May 2023 | Live performance + installation elements | Approx. 75 | Included coral/choir poetry cycle and tree–voice compositions. |
| **Way Out TV (Prison Broadcasting Service)** | UK National Network | 2023 (multiple screenings) | Broadcast screening | Estimated reach: 65,000 incarcerated viewers | A 20-minute edit of the project was broadcast several times upon request from the prison community. |

Total estimated reach:

- **875 in-person attendees**
- **~75,000 virtual viewers** (Way Out TV and internal community re-broadcasts)

A non-public preview video is available to editors on request.

## **2. Informal Audience Feedback**

Across venues, feedback described the work as:

- *“emotionally resonant, uplifting, and unexpected”*
- *“a novel merging of art, science, and survivorship”*
- *“profoundly moving to hear trees and people who both survived radiation”*
- *“a powerful demonstration of resilience”*

Audience members highlighted:

- the emotional impact of projected tree imaging
- the uniqueness of hearing survivor-tree recordings
- the visibility given to laryngectomy voices
- the therapeutic quality of the combined soundscapes

This informal feedback reinforced the thematic findings of awe, empathy, and shared survivorship.

## **3. External Artistic Collaborations**

### **3.1 Integration into ballet and contemporary performance**

Recordings from the project’s survivor-tree sound database were incorporated into the ballet **“Fusion”**, collaboratively created by Harry Yeff (Reeps100) and Gadi Sassoon as a commission for the **Leipzig Opera House** (Germany).
 This represents a secondary artistic use of the project’s ecological sound material.

### **3.2 Voice gems and digital artworks**

Tree and choir recordings were used by:

- **Harry Yeff (Reeps100)** and
- **Trung Bao**

to produce a series of **digital “voice gems”** combining survivor-tree vibrations with laryngectomy vocal textures.

This work was **nominated for the Lumen Prize (2024)**, recognising innovation at the intersection of art, sound, and technology.

## **4. Media Coverage**

The project received international arts coverage, including a long-form feature published in **Al Jazeera (Arts & Culture, July 2023)** describing the creative and scientific processes behind the work.

(Full text available to editors on request; no active link provided here to comply with journal guidelines.)
