# Supplementary material for: From silence into song: an art–science collaboration with survivor trees and laryngectomy singers
Source: Front Psychol. 2026 Jan 30;16:1747218. doi: 10.3389/fpsyg.2025.1747218 (PMC12903274; doi:10.3389/fpsyg.2025.1747218)
Supplement: Supplementary file 2 [file Supplementary_file_2.docx]

# Supplement 2-4:

Supplement 2: Key quotes from participants, composers, writer, and visual artist

Supplement 3: Workshops overview

Supplement 4: Thematic map linking workshops, data sources, and experiential themes

# Supplement 2: Key illustrative quotations

This supplement collates example extracts from participants and project collaborators (composers, writer, visual artist) used to support the themes reported in the main manuscript. Extracts are presented by role and grouped under the theme they most directly illustrate. Where available, extracts should be attributed by participant ID in the main paper or in a separate participant table.

### 2.1 Participants (laryngectomy choir members)

Parallel survivorship & ecological metaphor

- “These trees survived an atomic explosion and still prosper — their resilience inspired us all.”
- “Laryngectomee or not, most of us will experience illness or trauma in our lives and need such treelike resilience to endure.”
- “The trees and we share the same radiation, but both of us are still here, still growing.”

Reclaimed agency, performance, and public presence

- “For a ‘voiceless’ person to convey emotion through altered speech was an act of reclamation.”
- “We’re in control — they are listening!”
- “Who would have thought of a voiceless choir singing along with voiceless trees?”
- “After performing, I felt my voice had done its job — it had resonated, connected, and given something meaningful to others.”

Collective embodiment & all sound as communication

- “Singing together brought elation and confidence — a lifting of spirit.”
- “Emergence proved that all sound can be a form of communication, whether it be a hum or click or tut.”
- “To hear our voices merge with the sound of the trees was enlightening and inspiring… when combined it all fell into place.”

Re-voicing identity and loving the ‘new’ voice

- “I thought the operation wouldn’t define me — but it did, and I fight it every day.”
- “After enjoying my experience with the choir, I love the new sound of my voice.”
- “I, as a ‘voiceless’ laryngectomee, could show that I had varied intonation and volume…”

Humour, dignity, and everyday coping

- “I was flabbergasted that trees made such sounds… I would have considered that person demented who told me trees could talk !”
- “Amazon driver tells me that I sound rough: “I don’t care — I got my package.”

### 2.2 Composers

Parallel survivorship & empathy through sound

- “It became easier to understand the trees after I better understood the voices of the choir.”
- “At first the weight of the project was overwhelming… once I immersed myself in the minute detail of the tree sounds, I was able to hear what they were saying musically.”

Project as living organism / collective embodiment

- “It was like nothing else I’d ever worked on… The whole project itself feels like it’s living, growing, constantly branching outwards in unexpected tangents.”
- “The friendships and connections made with the collaborators have had a profound impact on my personal and artistic life.”

Process, risk-taking, and ‘happy accidents’

- “Trial and error was a significant part of the process… staying open to happy accidents, trusting one’s gut and instincts.”
- “It was composing in the true definition of the word: putting things together.”

Audience takeaway

- “I hope they carry with them some of the strength and resilience of the choir and the trees… an appreciation for the power of nature, and an empathy for all those affected by radiation.”

### 2.3 Writer

Writing as service and empathy

- “It gave me a way to use my performance and writing skills for the good of others, with no lens put on myself.”

Deep listening and the value of silence

- “Listening and learning about the people in the program was the most meaningful part.”
- “You come face to face with the fact that the voice is a precious gift, as is the choice of silence.”

Poetry as bridge / returning participants’ messages

- “Poetry lets profound emotional energy transverse the human condition.”
- “I tried to re-inject their own messages back into the presentation — poetry works like that, especially spoken word.”

Witnessing participants’ impact

- “They were beautifully surprised at how impactful their contributions are on people.”
- “Every art piece shifts my perspective — including this one.”

### 2.4 Visual artist

Lifelong affinity with trees and parallel survivorship

- “I’ve had a deep affinity with trees since childhood… the uncertainty in the movement of branches and leaves, and the way they play with light.”
- “A Nagasaki survivor told me that soon these trees will be the only living witnesses of the bombs. That sense of presence is unforgettable.”
- “We uncovered the trees’ hidden voices, just as Thomas has helped the choir members rediscover theirs. Both were silent, and now they sing together in harmony.”

Revealing invisible vitality

- “I wanted to capture something of their powerful life-force, so I used infrared and military-grade thermal cameras to reveal the trees beyond the ordinary.”

Respect, possibility, and hope

- “Meeting the choir members was extraordinary. They are such an inspiring group of people.”
- “What Thomas has achieved with the choir is audacious — it sweeps away any sense of limitation imposed by disability.”
- “Ultimately, the experience reaffirmed that anything is possible — and that perceived limitations should never hold them back.”

# Supplement 3 – Workshop Structures and Creative Processes

## Overview of Co-Creative Workshops (Workshops 1–6)

This supplement summarises the six co-creative workshops that generated the multimodal dataset for From Silence into Song / Emergence. For each workshop, it lists the focus, core activities, principal outputs, and the analytic theme(s) most strongly supported.

Workshops involved laryngectomy choir members (Shout at Cancer) and an interdisciplinary team (poetry, composition, sound and image) to translate lived experience into musical, poetic, and audio-visual material.

| Workshop | Title | Aim / Focus | Core activities & methods | Main themes & creative outputs | Link to analytic themes |
| --- | --- | --- | --- | --- | --- |
| 1 | “Grown Out of My Old Voice” – Musical Co-Creation with Survivor Trees and the Laryngectomy Choir  TM, BS, CD, TR, SAC | To explore how lived experiences of speaking and singing after laryngectomy can be translated into musical material in dialogue with survivor-tree recordings, and to surface psychosocial themes (confidence, humour, “thick skin,” being underheard/overlooked, advocacy). | Group discussion of stage experiences and everyday communication; introduction of survivor-tree recordings; identification of key phrases (“grown out of my old voice,” “we make them listen,” “do it again, but better, not bitter”); improvisation of spoken/choral refrains and call-and-response sections with tree pulses; sharing everyday vignettes (e.g., public reactions, family life & pets). | Drafted a performance structure with four sections (opening personal motif, confident choral hook, testimonial weave, “still growing” coda); established core textual and musical hooks (“we’re in control — they are listening,” “grown out of my old voice,” “still growing”); articulated barriers to participation and role of the choir as advocacy rather than “therapy add-on.” | Parallel survivorship: underheard humans/trees as co-survivors. Reclaimed agency: “we make them listen,” stage confidence mapped onto daily life. Collective embodiment: shared build from “humble/natural” to “adrenaline/joy,” performing humour and stigma. |
| 2 | “Themes of Voice and Renewal” – Poetic Theme Exploration  TM, BS, SAC | To generate poetic material and shared metaphors for collaborative lyric-writing and spoken-word pieces about post-laryngectomy life, using sensory prompts and emotional exploration. | Warm-up using sensory prompts (“if your voice had a colour/texture/temperature today…”); six rotating “theme stations” (gratitude & acceptance; transformation & rebirth; listening & excavation; pop song re-imagining; partnership & humour); small-group writing; plenary reflection and mapping of recurring images/words; optional improvisation with simple musical/ambient textures under spoken lines. | Produced banks of phrases and couplets feeding later poems and songs (e.g., “I lost… / I gained…,” coral/choir imagery, “light at the end of the tunnel,” “Hey Mood” reworking of Hey Jude); consolidated six thematic strands that later became core poems (“Ode to the Larynx,” “The Lumen of the Tunnel,” “Tourist in My Own Home,” “Grown Out of It,” “Hey Mood,” “O Boy Could He Drone”). | Parallel survivorship: nature and body as shared landscapes of recovery. Reclaimed agency: reframing irreversible change through gratitude, humour, and self-definition. Collective embodiment: coral/choir imagery of many small voices forming one protective whole. |
| 3 | “Now We Are Talking” – Reclaiming the Language of Connection  TM, BS, CD, TR, SAC | To critically and playfully rework telecom advertising slogans about connection in light of the slower, effortful reality of post-laryngectomy communication, and to combine these with survivor-tree sounds. | Listening to survivor-tree recordings and discussing “connection” (static, interference, “a line trying to connect”); group selection and critique of telecom slogans (“communication is life,” “be heard,” “life’s for sharing,” “can you hear me now?”); creation of poetic collages juxtaposing slogans with participants’ own lines (“nothing, nothing, absolutely nothing,” “do it again, but better, not bitter”); recording personal “call signs” (short self-authored mottos); rhythmic poem based on “the apple doesn’t fall far from the tree”; final layered improvisation blending slogans, personal lines and tree sounds. | Generated text and sound material for the piece Now We Are Talking; created a layered sound-poem combining tree pulses, telecom language, and laryngectomy voices; produced a bank of personal mottos (“listening is active, listening is positive,” “personally, I’m improving”) and the “apple/tree” rhythm poem. | Parallel survivorship: trees as “true communicators” and kin in a world of over-wired disconnection. Reclaimed agency: subverting corporate slogans into activist statements; asserting “we’re still on the line.” Collective embodiment: ensemble canon of overlapping slogans and voices mirroring shared struggle with noise, repetition, and mishearing. |
| 4 | “Each Story: Promise and Delivery” – Tree-Led Composition  TM, BS, CD, PC, SAC | To co-create a four-part musical structure in which survivor-tree recordings and laryngectomy voices mutually shape rhythm, texture, and emotional narrative, interrogating the gap between communication “promises” and lived reality. | Open-text score and improvisation with tree and fungi field recordings (hydrophone, accelerometer) as primary “score”; ensemble response on voice, percussion, and guitar; development of a “telecom tree canon” aligning telecom slogans with tree-derived speech rhythms; introduction of a diatonic choral song section symbolising regrowth; creation of a reflective coda with humming, breath sounds, theremin, and spoken social-reintegration phrases. | Established the four-movement structure Each Story: Promise and Delivery (I: Tree resonance; II: Telecom tree canon; III: Song of growth; IV: Coda/return); refined musical use of key phrases (“do it again, but better, not bitter”); integrated graft/regeneration imagery linking surgical grafts and tree bark; foregrounded “listening as art.” | Parallel survivorship: explicit linking of graft, regrowth, and survival in trees and humans. Reclaimed agency: reclaiming over-promising telecom language with truthful, co-authored sound. Collective embodiment: choir, ensemble, and trees forming one “musical ecology” where listening and synchrony are central. |
| 5 | “Finding New Words, Finding New Sound: From Coral to Choir” – Poetry and Lyric Workshop  TM, BS, CD, SAC | To translate experiences of voice loss and recovery into collaboratively authored poems and lyrics, using coral/choir and tree metaphors to explore identity, family, care, and humour. | Group discussion about life after laryngectomy (work, retirement, caring roles, family adjustments); prompts linking survivor trees and coral reefs to collective voice; collaborative word lists on fear, unexpected changes, caring realities; chained drafting of poems (participants → poet refinement → group feedback); re-writing of Hey Jude into “Hey Mood (Stay True)”; development of “coral” as visual and conceptual motif (e.g., “Join the Coral,” “We are Coral”); reading poems aloud and recording them with improvised choral textures. | Created six core poems: “O Boy Could He Drone,” “Hey Mood (Stay True),” “The Lumen of the Tunnel,” “Tourist in My Own Home,” “Grown Out of It,” and “Ode to the Larynx.” These became central textual sources for performances and for the qualitative analysis (identity, humour, fear, resilience, gratitude). Developed “Coral Choir” imagery and language as a unifying performance motif. | Parallel survivorship: coral and trees as analogues for choir – many small units sustaining a living whole. Reclaimed agency: participants as poets/bards, not patients; humour and philosophical reflection as tools for self-definition. Collective embodiment: coral/choir metaphor of interdependent voices and shared protective structures. |
| 6 | “From Silence into Song: Emergence” – Voice Beyond Voice  TM, HY, PC SAC | To develop Emergence, a multimedia work in which laryngectomy voices and survivor-tree sounds are fused through graphic scores, beatboxing, and infrared/thermal imagery, reframing voice as energy and vibration. | Beatboxing and breath-based exercises; introduction of graphic/visual metronomes guiding small-group vocal cycles; individual phone recordings following guide tracks (bright vs grounded takes); filming participants with infrared and thermal cameras from multiple angles; layering tree recordings with vocal textures; reflection on hearing oneself within a larger “choir” of humans and trees. | Produced the 3-minute piece Emergence (audio-visual score combining laryngectomy vocal textures, survivor-tree recordings, breath and stoma percussion, and environmental frequencies, with thermal/IR imagery of trees and performers); articulated shared key words/gestures around resilience and connection; reinforced “voice beyond speech” as a central project concept. | Parallel survivorship: explicit dialogue between radiated bodies (trees and humans) as co-survivors. Reclaimed agency: participants’ altered voices and gestures as core sonic material, not an afterthought. Collective embodiment: emergent chant-like structure where individual contributions form a single rising pulse of human–nature synchrony. |

## Supplement 4. Thematic map linking workshops, data sources, and experiential themes

This supplement shows how the themes reported in the Results were developed across workshops and data sources in From Silence into Song and Emergence.

See Methods for analytic approach (Braun & Clarke, 2006; 2019). The thematic map draws on the following sources:

- Workshop discussions and transcripts (Workshops 1–6)
- Creative artefacts (poems, lyrics, open text scores)
- Field notes and facilitator reflections
- Artist interviews (composer, writer, visual artist)
- Audio–visual documentation of rehearsals and performances

Table S4.1 maps (a) core themes and subthemes, (b) contributing workshops and project phases, (c) primary data sources, and (d) brief analytic notes on convergence.

### Table S4.1. Thematic map linking workshops, data sources, and experiential themes

| Core experiential theme | Subthemes / related codes | Key workshops / project phases | Primary data sources | Analytic notes (how theme emerged) |
| --- | --- | --- | --- | --- |
| 1. Parallel Survivorship | – Trees and people as co-survivors of radiation  – Radiation as paradox (harm/heal)  – Silence redefined as vitality, not absence  – Awe, wonder, and ecological metaphor | – W1 “Grown Out of My Old Voice” (initial linking of tree survival and laryngectomy journeys)  – W3 “Now We Are Talking” (telecom / tree canon)  – W4 “Each Story: Promise and Delivery” (tree recordings as “teachers”)  – W6 Emergence (tree + choir as shared energy field) | – Participant reflections on hearing tree recordings for the first time (group discussions, field notes)  – Poems and texts (“The Lumen of the Tunnel”, “Grown Out of It”, “Tourist in My Own Home”)  – Visual artist reflections on filming survivor trees  – Composer interviews describing “understanding the trees through the choir” | Participants repeatedly linked the endurance of the Hibakujumoku to their own survival after radiotherapy and laryngectomy (“the trees and we share the same radiation, but both of us are still here, still growing”). Hearing crackles, rumbles, and vibrations recast silence as evidence of hidden life. Artist accounts reinforced this, describing trees as “living witnesses” and imaging techniques as revealing an unseen life-force. Across workshops and Emergence, tree sounds and imagery functioned as a metaphorical mirror through which participants re-narrated their own survivorship. |
| 2. Reclaimed Agency | – Performance as reclamation of voice and public presence  – Confidence, responsibility, advocacy (“we make them listen”)  – Technology as extension of self (speech button, microphones, imaging)  – Humour and storytelling as control strategies | – W1 (stage feelings; “we’re in control – they are listening!”)  – W3 (reclaiming telecom slogans: “Communication is life”, “Be heard”)  – W4 (Promise and Delivery – over-promising slogans vs lived effort)  – W5 (poetry workshop: “O Boy Could He Drone”, “Ode to the Larynx”)  – Live performances (World Choir Games, Bloomsbury, ROSL, Lincoln, Way Out TV) | – Participant feedback on performing with altered voices  – Everyday vignettes (Amazon driver, Halloween, clinic, family humour)  – Co-created poems and rewritten pop lyrics (“Hey Mood / Stay True”)  – Composer and writer reflections on co-authorship and “using art in service of others”  – Field notes on rehearsal dynamics (hesitancy → leadership) | This theme captures movement from “patient” to agentic performer and advocate. Participants described feeling “natural” on stage, “alive, dynamic, responsible”, while also acknowledging anxiety and the risk of “making the wrong sound”. Phrases such as “we’re in control – they are listening!” and “we make them listen” became both musical hooks and analytic anchors. Humorous accounts (scaring kids at Halloween, joking with nurses, Amazon deliveries) were coded as humour-as-coping, showing how participants reclaimed potentially stigmatising moments. Artists and facilitators noted increased leadership and visibility in rehearsals and performances, reinforcing agency as both a psychosocial and performative achievement. |
| 3. Collective Embodiment | – Choir as social organism / coral reef  – Breath and rhythm synchrony  – Shared emotional “lift” and joy  – Human–nonhuman co-sounding (choir + trees)  – Listening as active, patient, mutual | – W1 (choir as uplifting: “we are entertaining, we are educating”)  – W2 (coral / choir metaphor: “Join the Coral”, “We are Coral”)  – W4 (four-part structure of Each Story: Promise and Delivery)  – W5 (poem cycle performed in ensemble)  – W6 Emergence (group metronomes, voice cycles, IR/thermal filming) | – Group descriptions of singing together (“elation and confidence – a lifting of spirit”)  – Coral / reef imagery and poems (“We are Coral”)  – Thermal and IR video showing shared breath and energy  – Emergence recording sessions (layered metronome-guided vocal takes)  – Field notes on mutual respect and turn-taking | Collective embodiment crystallised where bodies, breath, and sound aligned. Participants spoke of becoming part of a team “where everyone has a role and it all falls into place”. The coral metaphor framed the choir as a living protective structure made of individual “polyps” (voices). Technological work (tree pulses, coral colours, IR breath imaging, Emergence metronomes) made bodily synchrony visible and audible. This theme spans physical synchrony, emotional “lift”, and ethical attention to one another’s pace and effort in communication. |
| 4. Re-voicing Identity (feeds particularly into Themes 1 & 2) | – Continuity with past roles (teacher, musician, veteran, etc.)  – Negotiating “disability” vs “inconvenience”  – Growing into a “new voice” (“I’ve grown out of my old voice”)  – Intergenerational and family resonance | – W1 (“grown out of my old voice” hook)  – W2 (identity-focused stations: Transformation & Rebirth; Listening & Foreign Body)  – W5 (poems “Grown Out of It”, “The Lumen of the Tunnel”, “Tourist in My Own Home”, “Ode to the Larynx”)  – Artist interviews describing changed listening and identity | – Participant narratives about earlier performance or teaching careers  – Poems explicitly reflecting on old vs new voice  – Family reflections (granddaughter’s poem; “your journey is our journey”)  – Composer and writer accounts of how the project reshaped their own identity as artists | This theme traces how participants and artists negotiated who they are now. Participants contrasted their “old voice” with the “new sound” they have learned to appreciate, often with ambivalence (“I miss my old voice… although it was better, I’ve grown out of it”). The poetry workshop generated particularly dense material on identity, using images of tunnels, tourism in one’s own body, and growth. Artist reflections (composers, writer, visual artist) echoed this, describing shifts in how they understand voice, silence, and their own daily listening. Analytically, Re-voicing Identity functions as a bridge between Parallel Survivorship (recognising survival) and Reclaimed Agency (acting from that renewed self). |
| 5. Humour, Dignity, and Everyday Coping (supports Themes 2 & 3) | – Family/playful humour (“Nanny-Oooh”)  – Public encounters (delivery drivers, nurses, airports)  – Laughing at leakage, button-pressing, coughing  – Normalising altered voice | – W1 (“thick skin” / speech button as filter)  – W3 (irony with telecom slogans)  – W5 (“O Boy Could He Drone” and other humorous texts)  – Ongoing rehearsal anecdotes and informal feedback | – Everyday vignettes collected in workshops and field notes  – Humorous poems and lines performed in concert  – Audience feedback noting both poignancy and lightness | Humour appeared consistently as a dignifying strategy, allowing participants to hold vulnerability without being defined by it. Stories about grandchildren, delivery drivers, clinic interactions and public “mishaps” (leaks, coughing) were told with wit and warmth. In analysis, these were not treated as “side stories” but as central evidence of adaptive coping, supporting themes of Reclaimed Agency (taking control of the narrative) and Collective Embodiment (shared laughter strengthening group bonds). |
| 6. Rediscovering the Body & Sensory Adaptation (supports Themes 1 & 3) | – “Tourist in my own home” – body as new landscape  – Listening as healing practice  – Attention to breath, stoma, vibration  – Slowing down; altered tempo of life | – W2 (station “Listening & the Foreign Body”)  – W5 (“Tourist in My Own Home”)  – W6 Emergence (voice as energy field; IR/thermal filming) | – Poetic imagery of a foreign-yet-welcoming bodily landscape  – Group reflections on new ways of listening to self, others, and trees  – Visual artist’s language around capturing inner “life-force”  – Field notes on breathing exercises and sensory focus | This theme captures the embodied recalibration following laryngectomy: new airways, changed pressures, different sensory cues. Creative exercises explicitly asked participants to imagine their body as a new country or terrain. Emergence further externalised this process by visualising warmth and breath as glowing patterns. Analytically, this theme underpins Parallel Survivorship (body as “scarred but alive”) and Collective Embodiment (shared breath and attentive listening). |
| 7. Resilience, Renewal, and Strength (integrative / cross-cutting) | – Seeds, grafts, regrowth  – Continuing to grow despite trauma  – “Spreading seeds and letting it grow”  – Resilience as shared human–ecological rhythm | – W1 (seeds / “still growing” motif)  – W4 (tree graft, promise vs delivery)  – W5 (poem cycle, especially “Grown Out of It”)  – W6 Emergence (from silence to collective pulse) | – Participant phrases about seeds, growth, life after radiation  – Performance reflections (“we’re doing this for a bigger reason”)  – Artist accounts of the project as “living, growing, branching outwards”  – Audience feedback noting hope and uplift | This integrative theme describes resilience not as static toughness but as ongoing renewal. Seeds, coral, grafts, tunnels, and trees all served as repeated metaphors for enduring and continuing to grow after trauma. Participants connected their own survival to broader patterns of regeneration in nature. Artists likewise described the work as “living” and constantly evolving. In the analytic process, this theme sits behind all others, framing the project as a shared act of recovery across human and non-human voices. |
