# Supplementary material for: From silence into song: an art–science collaboration with survivor trees and laryngectomy singers
Source: Front Psychol. 2026 Jan 30;16:1747218. doi: 10.3389/fpsyg.2025.1747218 (PMC12903274; doi:10.3389/fpsyg.2025.1747218)
Supplement: Supplementary file 3 [file Supplementary_file_3.docx]

## Supplement 5-8: Reflexive Thematic Analysis

Supplement 5: Reflexive Thematic Analysis – Laryngectomy Choir Perspectives

Supplement 6: Reflexive Thematic Analysis – Visual Artist Perspective

Supplement 7: Reflexive Thematic Analysis – Composer Perspectives

Supplement 8: Reflexive Thematic Analysis – Writer Perspective

## Supplement 5: Reflexive Thematic Analysis: Laryngectomy Choir Perspective

This supplement provides an audit trail for the reflexive thematic analysis reported in the main manuscript. It summarises (i) codes and subthemes, (ii) example extracts, and (iii) analytic notes recorded during theme development. Extracts are verbatim and are attributed by role (participant/composer/writer/visual artist) and, where available, by participant ID. Analytic notes distinguish between observations recorded during workshops and later interpretive reflections. See Methods for analytic approach (Braun & Clarke, 2006; 2019).

Table S5.1. Reflexive thematic analysis – participant (choir) perspective

| **Theme** | **Codes / subthemes** | **Illustrative participant quotes** | **Facilitator / researcher notes** |
| --- | --- | --- | --- |
| **1. Parallel Survivorship** | Shared endurance; radiation as paradox; silence redefined as vitality; nature–human analogy | “I was flabbergasted that trees made such sounds — I had always thought that trees were inert.”  “These trees survived an atomic explosion and still prosper — their resilience inspired us all.”  “The trees and we share the same radiation, but both of us are still here, still growing.”  “Any living organism fights to survive despite all odds.”  “Calming and unified — similar to sitting under a tree listening to wind in the leaves.”  “It sounded like something snapping under pressure — like bats using echolocation.”  “There may seem to be no life, but beneath the scarred surface, there is a will to survive.”  “Personally I think all survivors of any life-altering event could feel a connection to the trees’ survival.” | [TM] Participants drew symbolic parallels between their survival and that of the Hibakujumoku; silence emerged as evidence of vitality, not absence. [IR] Tree imagery highlighted radiation as both trauma and regeneration; “treelike resilience” became a shared metaphor for recovery. |
| **2. Reclaimed Agency** | Public performance; confidence and control; humour; empowerment; training the audience to listen | “For a ‘voiceless’ person to convey emotion through altered speech was an act of reclamation.” “We’re in control — they are listening!” “You have to train the audience — they do more effort; their listening improves.” “It feels natural for me to be on stage: I can make up for the loss of my voice box.” “It’s all about helping others regain confidence.” “So proud to be part of the choir — to make a difference for anyone who has to go through the same thing.” “Natural — a bright way to make up for the loss of vocal cords.” “Elevation, achievement, self-worth, I have meaning.” “Adrenaline, joy, alive, dynamic, responsible for the community.” “Frightening to make a wrong sound — but I’m in control!” “One day you’ll get that ‘Oh, that’s it!’ feeling.” | [IR] Agency grew through public performance; participants positioned themselves as educators and advocates. [TM] Intelligibility was reframed as more important than vocal tone; the audience became an active partner in communication. [TM/IR] Extracts also described a shift from apprehension to confidence during performance. |
| **3. Collective Embodiment** | Choir as social organism; mutual respect; shared rhythm; attuned listening; human–nonhuman harmony | “In our group there is mutual respect — we let each other speak.”  “Singing together brought elation and confidence — a lifting of spirit.”  “Emergence proved that all sound can communicate, whether hum, click, or tut.”  “Not voices as such — more a harmonic connection between nature and ourselves.”  “The music felt calm and unified.”  “To hear our voices merge with the trees was enlightening and inspiring.”  “If you break sound to its base level, we can harmonise and uplift.”  “As soon as I joined, I became part of a team; everyone has a role and it all falls into place.” | [TM] Breath synchrony and mutual attention strengthened group cohesion. [IR] Co-sounding with trees revealed shared rhythm and shared rhythm. |
| Cross-cutting subtheme: Re-voicing identity | Continuity with past self; acceptance; adapting through artistry; growth mindset | “I thought the operation wouldn’t define me — but it did, and I fight it every day.”  “After enjoying my experience with the choir, I love the new sound of my voice.”  “I found the song cathartic — it reminded me of the loss of my voice and of song.”  “It is not a disability — it’s an inconvenience that didn’t take half measures.”  “I’ve accepted that I’ll sometimes be understood, sometimes mute.”  “It has become a filter — I don’t feel constipated about anything anymore.”  “You learn to be positive; small things with a huge impact make you stronger — you are growing.” | [IR] Identity reconstruction was active, embodied, and often humorous. [TM] “Voice as filter” became a metaphor for selective communication and new self-definition. |
| Cross-cutting subtheme: Intergenerational resonance | Family influence; modelling resilience; emotional contagion | “My severely dyslexic granddaughter wrote me a poem — she overcame the same boundary.”  “Your journey is our journey.” | [TM] Reflections showed ripple effects of pride, family healing, and mirrored resilience. |
|  |  |  |  |
| Cross-cutting subtheme: Humour, dignity, and everyday coping | Family humour; public interactions; reclaiming awkward situations | “My granddaughter calls me Nanny-Oooh.”  “Amazon driver says I sound rough; I don’t care — I got my package.”  “Halloween scaring the kids with the stoma.”  “The nurse cracked up when I said I was joining a choir — best excuse ever.” | [TM] Humour diffused stigma and supported dignity in daily interactions. |
| Cross-cutting subtheme: Body and sensory adaptation | Listening as healing; slowing down; new sensory calibration | “Listening has a healing aspect; I can slow sounds down.”  “Calming and unified — like sitting under a tree.”  “Tourist in my own home.” | [IR] Participants described sensory and perceptual recalibration post-surgery. [TM] Tree soundscapes supported interoceptive calm and grounding. |
| Cross-cutting subtheme: Technological resonance | Technology as defining voice; vibrations as communication | “Technology like contact mics broadened my idea of what a voice can be.”  “Who would have thought of a voiceless choir singing with voiceless trees?”  “The technology showed that things we think are silent are communicating.”  “By changing how we listen, we open the door to communication we never thought possible.” | [IR] Technology became a creative extension — expanding the ontology of “voice.” [TM] Scientific listening revealed hidden vitality and deepened the art–science dialogue. |
| Cross-cutting code cluster: Resilience, renewal, and strength | Endurance; growth after trauma; performance as renewal | “You can only spread the seeds and let it grow — hope something comes out.”  “We’re doing this for a bigger reason.”  “I would never have thought I’d experience anything so uplifting.”  “Life is beautiful — it never stopped me going forward.”  “Laryngectomee or not, we all need treelike resilience to endure.”  “I could show that I had varied intonation and volume.”  “The trees’ survival showed that strong will overcomes obstacles.”  “The audience could sense the shared stories of healing and rejuvenation.” | [TM] Narratives reframed survival as creative thriving. [IR] The “tree” metaphor became a lived symbol of regeneration and embodied hope. |

Reflexive commentary (S5)
 [TM] The integrated reflections reveal how sonic, artistic, and emotional discovery became inseparable. Participants repeatedly used the trees as mirrors for their own recovery, and the workshops blurred boundaries between therapy, performance, and meaning-making. Technology functioned not as an add-on but as revelation, expanding what “voice” could be.
 [IR] What stands out is the shift from novelty (“trees make sounds?”) to kinship (“they survived like us”). Listening became a mode of knowing, healing, and belonging. Voice, vibration, and silence all operated as forms of communication—allowing participants to construct resilient, interconnected identities.

## Supplement 6. Reflexive Thematic Analysis – Visual Artist Perspective

This supplement provides an audit trail for the reflexive thematic analysis reported in the main manuscript. It summarises (i) codes and subthemes, (ii) example extracts, and (iii) analytic notes recorded during theme development. Extracts are verbatim and are attributed by role (participant/composer/writer/visual artist) and, where available, by participant ID. Analytic notes distinguish between observations recorded during workshops and later interpretive reflections. See Methods for analytic approach (Braun & Clarke, 2006; 2019).

### Table S6. Reflexive Thematic Analysis – Visual Artist Perspective

| Theme | Codes / Subthemes | Illustrative Artist Quotes | Facilitator / Researcher Notes |
| --- | --- | --- | --- |
| Embodied Affinity with Nature | Lifelong connection to trees; movement, light, and mystery as creative stimulus | “I’ve had a deep affinity with trees since childhood… the uncertainty in the movement of branches and leaves, and the way they play with light.” | [TM] The artist’s naturalist lens offers continuity between organism and observer.  [IR] Affinity here acts as grounded empathy — perception as relational attunement. |
| Parallel Survivorship (Nature and Humanity) | Trees as witnesses; endurance and regeneration; shared trauma and healing | “A Nagasaki survivor told me that soon these trees will be the only living witnesses of the bombs.”  “We uncovered the trees’ hidden voices, just as Thomas has helped the choir members rediscover theirs. Both were silent, and now they sing together in harmony.” | [TM] The visual metaphor of parallel survivorship mirrors the medical narrative of recovery. [IR] Dual witnessing — the camera becomes a mediating role between trauma and vitality. |
| Revealing the Invisible | Infrared and thermal vision; making energy visible; translating unseen vitality | “I wanted to capture something of their powerful life-force, so I used infrared and military-grade thermal cameras to reveal the trees beyond the ordinary.” | [TM] The use of thermal imaging mirrors diagnostic seeing — a bridge between artistic and medical technologies. [IR] This is sensory attention: re-perceiving vitality through altered vision. |
| Voice, Silence, and Presence | Sound as revelation; trees as communicators; temporal witnessing | “To record their ‘voices’ at the exact time of the atomic explosions, 75 years on, was very special.” “Hearing those crackles, pops, rumbles and thumps — the inner workings of the trees — was extraordinary.” | [TM] Recording sound at the exact historical moment reclaims silence as commemoration. [IR] Temporal resonance functions as ritual — sound bridges time and trauma. |
| Human–Nonhuman Connection | Choir and trees as co-witnesses; shared resilience; coexistence of silence and song | “Meeting the choir members was extraordinary. They are such an inspiring group of people.” “We uncovered the trees’ hidden voices, just as Thomas has helped the choir members rediscover theirs.” | [TM] The artist perceives parallel courage between laryngectomees and trees. [IR] relational approach: the artwork becomes a cross-species empathy field. |
| Re-voicing Hope and Possibility | Overcoming limitation; renewal through creation; spiritual resilience | “What Thomas has achieved with the choir is audacious — it sweeps away any sense of limitation imposed by disability.” “That anything is possible — and that perceived limitations should never hold them back.” | [TM] Optimism is framed as active agency — turning adversity into invitation. [IR] The visual artist adopts the stance of hopeful witnessing. |
| Witnessing as Transformation | Documentary ethics; emotional transmission; art as healing testimony | “Meeting the choir members was extraordinary. They are such an inspiring group of people.” “Filming in Hiroshima and Nagasaki was deeply moving… a spiritual encounter with survival.” | [TM] The camera becomes an instrument of reverence and care. [IR] Filming functions as relational witnessing — presence replaces distance. |

### Reflexive memo extracts (TM/IR)

• TM memo: Infrared/thermal imaging functioned as a way of noticing vitality; filming was treated as witnessing rather than extraction.

• IR memo: Recording at the hour of the blasts introduced a commemorative frame; sound and image were used to hold memory alongside renewal.

• TM/IR memo: The artist’s long-standing affinity with trees shaped an empathic stance; silence was repeatedly discussed as presence rather than absence.

## Supplement 7. Reflexive Thematic Analysis – Composer Perspectives

This supplement provides an audit trail for the reflexive thematic analysis reported in the main manuscript. It summarises (i) codes and subthemes, (ii) example extracts, and (iii) analytic notes recorded during theme development. Extracts are verbatim and are attributed by role (participant/composer/writer/visual artist) and, where available, by participant ID. Analytic notes distinguish between observations recorded during workshops and later interpretive reflections. See Methods for analytic approach (Braun & Clarke, 2006; 2019).

### Table S7. Reflexive Thematic Analysis – Composer Perspectives

| Theme | Codes / Subthemes | Illustrative Composer Quotes | Facilitator / Researcher Notes |
| --- | --- | --- | --- |
| Parallel Survivorship (via composer empathy) | Translating survival into sound; radiation as paradox (harm/heal); understanding trees through understanding the choir | “It became easier to understand the trees after I better understood the voices of the choir.” “At first the weight of the project was overwhelming… once I immersed myself in the minute detail of the tree sounds, I was able to hear what they were saying musically.” | [TM] Composers mirrored participants’ emotional processing through sound. [IR] Empathy operates here as both an aesthetic and psychological method — listening becomes understanding. |
| Reclaimed Agency (artistic) | Trusting process; ‘happy accidents’; iterative risk-taking; co-authorship | “Trial and error was a significant part of the process… staying open to happy accidents, trusting one’s gut and instincts.” “Working with quite abstract material… towards the end it felt like we had developed our own language to describe different elements of the piece.” | [TM] Agency expressed through uncertainty tolerance — ‘trusting the process’ mirrors vocal recovery. [IR] Co-composition itself was restorative: uncertainty recast as creative vitality. |
| Collective Embodiment | Project as living organism; social flow; relational composition | “It was like nothing else I’d ever worked on… The whole project feels like it’s living, growing, branching outwards in unexpected tangents.” “The friendships and connections made with the collaborators have had a profound impact on my personal and artistic life.” | [TM] The project itself “breathed” — a living extension of interdependence. [IR] Their language of vitality (‘living’, ‘growing’) evokes distributed authorship and psychosocial synchrony. |
| Re-voicing Identity (composer reflexivity) | Changed daily listening; voice redefined; identity shift through proximity to altered voice | “My approach to the voice, even in daily life, has been deeply impacted.” “Getting to know the choir and understand their experiences has been transformative, both socially and musically.” | [TM] Artistic identity softened and expanded through medical empathy. [IR] Reflexive turning point: the artist becomes listener; authorship is redefined through relation rather than mastery. |
| Technological Resonance | Tools as empathy; detail-first listening; balancing layers | “Once I immersed myself in the detail of the tree sounds, I could hear what they were saying musically.” “Between Phil’s soundscapes, my melodies, and harmonies built around tree rhythms… it was a real balancing act to get all these layers working together.” | [TM] Technology functioned as both stethoscope and canvas — translating vibration into story. [IR] Mediated listening becomes knowledge-related: technology acts as co-creator in the phenomenology of sound. |
| Compositional Weave | Putting disparate materials together; flexibility in performance; co-design with collaborators | “It was composing in the true definition of the word: putting things together.” “We generated a lot of material very quickly… it’s quite a different piece each time it’s performed.” | [TM] Flexibility reflected the biological metaphor — always adapting. [IR] Structural fluidity mirrored emotional resilience; the composition remained alive to context. |
| Holding the Weight (affect balance) | Narrative heaviness vs infectious lightness; joy as resilience | “The weight of the narrative can be overbearing. But the infectious lightness of everyone involved has been more impactful.” | [TM] Balancing gravity and humour sustained the ensemble’s spirit. [IR] Joy is recast as affective intelligence — a form of balance through shared creativity. |
| Audience Takeaway (composer intent) | Strength, empathy, reverence; destruction–healing dialectic | “I hope they carry with them some of the strength and resilience of the choir and the trees… an appreciation for the power of nature, and an empathy for all those affected by radiation.” | [TM] The composers’ aspirations extended clinical empathy to audiences. [IR] Audience reception framed as emotional transmission — art as transmission of resilience. |

### Reflexive memo extracts (TM/IR)

• TM memo: The composers’ language (“living”, “growing”, “balancing”) echoed rehabilitation metaphors without being reduced to them; adaptation was a shared concern.

• IR memo: Openness to uncertainty and iteration (“happy accidents”) operated as a creative parallel to learning a new vocal system; authorship became distributed.

• TM/IR memo: Listening was repeatedly foregrounded as method—technical, relational, and ethical—rather than as a purely musical skill.

## Supplement 8: Reflexive Thematic Analysis – Writer Perspective

This supplement provides an audit trail for the reflexive thematic analysis reported in the main manuscript. It summarises (i) codes and subthemes, (ii) example extracts, and (iii) analytic notes recorded during theme development. Extracts are verbatim and are attributed by role (participant/composer/writer/visual artist) and, where available, by participant ID. Analytic notes distinguish between observations recorded during workshops and later interpretive reflections. See Methods for analytic approach (Braun & Clarke, 2006; 2019).

### Table S8. Reflexive Thematic Analysis – Writer Perspective

| Theme | Codes / Subthemes | Illustrative Writer Quotes | Facilitator / Researcher Notes |
| --- | --- | --- | --- |
| Purpose and Service | Writing as empathy; art in service of others; altruistic creativity | “It gave me a way to use my performance and writing skills for the good of others, with no lens put on myself.” | [TM] The writer frames authorship as care work — translating observation into dignity. [IR] Writing recast as empathy aligns with therapeutic storytelling models. |
| Deep Listening | Listening beyond words; witnessing gesture and silence; vocal fragility as truth | “Listening and learning about the people in the program was the most meaningful part.” “You come face to face with the fact that the voice is a precious gift, as is the choice of silence.” | [TM] Listening here mirrors the clinician’s stance in rehabilitation: patience, attunement, presence. [IR] The writer performs ethnographic listening — silence recognised as both data and meaning. |
| Voice and Connection | Revaluing communication; voice as conduit, not outcome; shared humanity | “The world tends to concentrate too much on the final sonic result and not on the connection, conduit, or contact it produces to the audience.” | [TM] The writer echoes a key therapeutic shift: communication as relational, not performative. [IR] Art is recast as dialogue over display — connection replaces performance as the metric of success. |
| Poetry as Bridge | Language as mirror; re-injecting meaning; emotional transference through text | “Poetry lets profound emotional energy transverse the human condition.” “I tried to re-inject their own messages back into the presentation — poetry works like that, especially spoken word.” | [TM] Poetry becomes a therapeutic feedback loop — returning participants’ truths through rhythm and form. [IR] Text functions as a relational object, mediating identity reconstruction and holding emotion. |
| Witnessing Transformation | Recognising participant impact; reflective awe; reciprocal growth | “They were beautifully surprised at how impactful their contributions are on people.” “Every art piece shifts my perspective — including this one.” | [TM] The writer acts as co-witness to growth, mirroring the clinician’s experience of transformation through others. [IR] Reflexive empathy loop — awareness of shared humanity reconfigures both observer and observed. |

### Reflexive memo extracts (TM/IR)

• TM memo: The writer consistently framed authorship as service; the work depended on patient listening rather than polished delivery.

• IR memo: Silence and gesture were treated as meaningful data; poetry was used to return participants’ meanings without claiming to ‘translate’ them.

• TM/IR memo: Re-injecting participants’ messages into the performance operated as reciprocity; the writer positioned themselves as conduit rather than interpreter.
