# Supplementary material for: From silence into song: an art–science collaboration with survivor trees and laryngectomy singers
Source: Front Psychol. 2026 Jan 30;16:1747218. doi: 10.3389/fpsyg.2025.1747218 (PMC12903274; doi:10.3389/fpsyg.2025.1747218)
Supplement: Supplementary file 4 [file Supplementary_file_4.docx]

This supplement provides the initial and final prompts supplied to the generative artificial intelligence tool (ChatGPT, OpenAI GPT-5.1) during manuscript preparation. In accordance with Frontiers’ policy, we limited AI input to prompts related to **language optimisation, structural clarity, and formatting**.

## Initial prompts

### **Prompt A – Language clarity and editing**

“Please help me refine the following paragraph for clarity and academic tone, without changing the scientific meaning.

### **Prompt B – Formatting a table**

“Format this material into a clear table suitable for a supplementary file, maintaining all original content and without introducing new interpretations.”

### **Prompt C – Organising supplementary materials**

“Reorganise the following workshop notes into a structured supplementary document, ensuring accuracy and preserving all participant-generated content.

### **Prompt D – Consistency editing**

“Please check this section for consistency in terminology, tense, and style, and provide a cleaned-up version that preserves the meaning.”

## Final prompts

### **Prompt E – Final proofreading**

“Perform a final proofread for grammar, syntax, and readability. Do not change scientific content.”

### **Prompt F – Formatting for Supplementary Material**

“Format this text as a clean supplementary table following academic conventions, without altering the underlying meaning.”

## **9.3 Statement of Compliance**

- These prompts were used solely for editorial purposes.
- All AI-edited sections were manually reviewed, corrected, and verified by the human authors.
- The use of ChatGPT (OpenAI GPT-5.1, OpenAI, USA) and Grammarly is acknowledged in the manuscript’s Acknowledgements section.
